# Visual cortical areas of the mouse: comparison of parcellation and network structure with primates

**DOI:** 10.3389/fncir.2014.00149

**Published:** 2015-01-07

**Authors:** Marie-Eve Laramée, Denis Boire

**Affiliations:** ^1^Laboratory of Neuroplasticity and Neuroproteomics, Department of Biology, KU Leuven-University of LeuvenLeuven, Belgium; ^2^Département d’anatomie, Université du Québec à Trois-RivièresTrois-Rivières, QC, Canada

**Keywords:** evolution, sensory pathways, feedforward, feedback, hierarchy, connectivity, cross-modal, connectome

## Abstract

Brains have evolved to optimize sensory processing. In primates, complex cognitive tasks must be executed and evolution led to the development of large brains with many cortical areas. Rodents do not accomplish cognitive tasks of the same level of complexity as primates and remain with small brains both in relative and absolute terms. But is a small brain necessarily a simple brain? In this review, several aspects of the visual cortical networks have been compared between rodents and primates. The visual system has been used as a model to evaluate the level of complexity of the cortical circuits at the anatomical and functional levels. The evolutionary constraints are first presented in order to appreciate the rules for the development of the brain and its underlying circuits. The organization of sensory pathways, with their parallel and cross-modal circuits, is also examined. Other features of brain networks, often considered as imposing constraints on the development of underlying circuitry, are also discussed and their effect on the complexity of the mouse and primate brain are inspected. In this review, we discuss the common features of cortical circuits in mice and primates and see how these can be useful in understanding visual processing in these animals.

## Is the mouse brain simple?

The mouse presents many advantages for the study of neural functions, circuits and their underlying genetic and molecular mechanisms. Its small size and ease of breeding offer significant advantages over the use of larger, less prolific and more costly housing and care of larger mammals. The mouse is a small mammal and a small rodent, and its brain is both small in absolute and in relative terms. An often represented bivariate log-log plot of brain size over body size clearly shows rodents to be in the most inferior portion of the minimum convex polygon for all mammals. The encephalization quotient of some of the smallest brained rodents is comparable to that of monotremes and marsupials (Striedter, 2004). The question here is to see whether the small size of the mouse brain also indicates its level of complexity. Is a small brain also a simpler brain?

Size has a particular significance in the evolutionary history of mammals because the earliest mammals emerged from particularly small ancestors and were not brainier than their reptilian ancestors (Kaas, 2011; Rowe et al., 2011). Throughout the evolution of mammals, an increase of the relative brain size has appeared independently in several groups, namely in primates, whales and dolphins and elephants. A great evolutionary radiation followed the initial increase of relative brain size, suggesting that more encephalized species were better at invading new niches or adaptive zones. In this respect, rodents appear to contradict this trend. With more than 2000 species and 30 different families, the order Rodentia is the most diverse order of placental mammals (Jansa and Weksler, 2004; Wilson and Reeder, 2005). It is quite stunning that over 40% of all mammalian species are rodents. They are found on all continents and exhibit a wide range of lifestyles from terrestrial, arboreal desert living, to aquatic, fossorial and even some achieve amazing feats of gliding flight. The range of body size varies more than 1000 fold and brain size by 200 fold. Yet, despite this tremendous adaptive radiation, the encephalization quotients of rodents are quite similar.

### Brain size and number of brain areas

The relationship between complexity and brain size is not clear cut. The general principle that larger brains are more complex is generally considered as fact. In their seminal comparative studies of brain size in Insectivores, Chiroptera and Primates, Stephan et al. considered that: “… increased size is almost always accompanied by progressive differentiation…” (Stephan et al., 1981). This view is challenged by an alternate hypothesis that proposed that: “…changes in the complexity of neural systems, in terms of the number of identifiable subdivisions, occur only during the evolutionary events leading to the establishment of a new mammalian order.” Therefore, within an order, all species should have the same organization of nuclear systems regardless of life history, brain size and time since evolutionary divergence (Manger, 2005). This hypothesis has been verified for the differentiation of cholinergic, cathecolaminergic and orexinergic nuclear masses in rodents (Kruger et al., 2012), visual cortical areas in carnivores, somatosensory and motor areas in primates and cortical areas in monotremes (Manger, 2005). This particular hypothesis questions the proposal that an increase in brain size necessarily leads to an increase in brain complexity. It implies that the higher levels of complexity of neural systems observed in the larger brains of primates would not be dependent on size but other factors. This hypothesis is interesting and should be further studied. As yet, there is no direct test and robust cladistic analysis of the relationship between brain size, either absolute or relative, and the complexity of the component neural systems.

There is another interesting corollary to this hypothesis. Considering that the mouse is amongst the smallest rodents, its brain would be neither more complex nor any simpler than other rodents regardless of the diversity of lifestyles and brain size. This does not mean that all rodents are identical, but proposes that they should have the same complement of nuclear masses and cortical areas. In this respect, a recent comparison of the cortical organization in several rodents representative of the main suborders, life history trait and levels of encephalization shows a general common pattern of neocortical organization, as well as the diversity of the relative size of the different sensory field and of the central magnification factors within these fields (Campi et al., 2007, 2011; Campi and Krubitzer, 2010; Krubitzer et al., 2011). This survey of rodent cortex shows a quite striking common set of cortical areas that can be found in numerous other orders of mammals. The authors do propose however several differences in the number of cortical areas in different species that would challenge the hypothesis of Manger (2005). For instance, although the ubiquity of the location and presence of the primary visual area in all rodents is not questioned, the number and parcellation scheme of extrastriate visual areas in rodents remains a matter of debate. There have been several attempts to decipher the organization of extrastriate cortices in the mouse (Wagor et al., 1980; Schuett et al., 2002; Van Der Gucht et al., 2007; Wang and Burkhalter, 2007; Garrett et al., 2014) and rat (Espinoza, 1983) as well as in a few other rodents (Thompson et al., 1950; Hall et al., 1971; Kaas et al., 1972; Tiao and Blakemore, 1976; Choudhury, 1978; Espinoza, 1983; Espinoza et al., 1992) and it is yet not clear that all rodents have the same complement of visual areas, as would require Mangers’ hypothesis.

### Organization of rodent visual areas

In the early literature, Rose had proposed that V1 is surrounded by at least five distinct visual extrastriate areas (Rose, 1929). However, there is no clear cytoarchitectonic differentiation of these areas lateral and medial to V1, and Caviness (1975) proposed that the primary visual cortex is flanked by only the two lateral and medial areas, 18a and 18b respectively. Tracing experiments have shown that V1 projects to several distinct sites in the cortices lateral and medial to V1 in mouse (Olavarria et al., 1982; Olavarria and Montero, 1989; Wang and Burkhalter, 2007; Wang et al., 2012) and rat (Montero et al., 1973). Electrophysiological mapping (Wagor et al., 1980) and optical imaging (Schuett et al., 2002) also suggest the presence of several medial and lateral extrastriate areas in the mouse, although the number and parcellation does not strictly correspond to the anatomical findings. In addition, neurofilament staining revealed delineation of monocular and binocular V1, in addition to two lateral and five medial extrastriate areas (Van Der Gucht et al., 2007). More recent anatomical and functional studies in mice provide quite convincing evidence for the presence of at least 9 extrastriate areas surrounding V1 in mice that exhibit distinct functional properties (Wang and Burkhalter, 2007; Andermann et al., 2011; Marshel et al., 2011; Roth et al., 2012; Wang et al., 2012; Glickfeld et al., 2013, 2014). Whether similar areas are also present in other rodents has not been adequately investigated. According to Mangers hypothesis, these visual areas would be very similar in all rodents. This hypothesis has yet to be thoroughly tested.

The comparison of mice and rats with squirrels is highly relevant. Squirrels are diurnal rodents and rely more on vision than the nocturnal muridae. In this respect, they have higher encephalization quotients and larger visual cortical areas than murids (Krubitzer et al., 2011). Anatomical (Kaas et al., 1989) and electrophysiological mapping (Hall et al., 1971) of the lateral extrastriate cortex in squirrels has led to the suggestion that there is one single visual field representation therein and more visual areas lateral to V2. This conclusion, in light of the more recent information in mice, is rather surprising in that it would suggest a less elaborate parcellation of visual cortical fields in a diurnal highly visual rodent than in a less visual nocturnal rodent. These results on the visual fields of the mouse therefore challenge the present understanding of the evolution of the visual cortex and of its organization.

In the present state of our understanding of the homologies between visual cortical areas in mammals, it is generally accepted that, in the initial mammals, there was a primary visual cortex located in the occipital region of the cortical sheet that appears to be common to all mammals, and that this V1 is flanked laterally by a single area V2 that would also be common to all mammals. This is the simple extrastriate cortex hypothesis (Rosa and Krubitzer, 1999). The opposing “complex hypothesis” states that V1 shares its lateral border and representation of the vertical meridian with multiple visual areas (Rosa and Krubitzer, 1999). The arguments opposing the simple and complex hypothesis have been exposed in detail in the review of Krubitzer on this specific subject and they will not be repeated here (Rosa and Krubitzer, 1999). We do believe however that some points should be reconsidered. The simple hypothesis is supported by the fact that a single representation of the visual field lateral to V1 and making up V2 is found in squirrels and that Sciuridae are considered as representative of the ancestral rodents (see Robinson et al., 1997; in Rosa and Krubitzer, 1999). The tracing of the V1 projections to lateral cortices in squirrels shows a patchy distribution of efferents (Kaas et al., 1989) not much different to what has recently been shown as indications of multiple extrastriate areas in mice (Wang and Burkhalter, 2007). This patchy distribution is presently interpreted, as in primates, to represent connection between related modules from V1 to V2 within a single visual field representation without notable discontinuities. Indeed, in monkeys, cytochrome dense blobs of V1 project to thin stripes in V2 (Livingstone and Hubel, 1983, 1984; Sincich and Horton, 2002, 2005; Sincich et al., 2007) and interblobs of V1 preferentially project to V2 thick and pale stripes (Xiao and Felleman, 2004; Sincich et al., 2010). The modular hypothesis for the visual projections to lateral V2 in squirrels is rather surprising given that there are no demonstrated modules in their visual cortex. There is no evidence for ocular dominance columns (Weber et al., 1977) and, although there are abundant orientation selective neurons, there are no orientation maps in the primary visual cortex of squirrels (Van Hooser et al., 2005a,b; Van Hooser and Nelson, 2006). In addition, the long range intrinsic connections within the primary visual cortex do not show a patchy distribution (Van Hooser et al., 2006) as is shown in mammals with modular visual cortices (Gilbert and Wiesel, 1983; Callaway and Katz, 1990; Malach et al., 1993; Ruthazer and Stryker, 1996; Bosking et al., 1997; Wang and Burkhalter, 2007). In mammals that exhibit functional maps, intrinsic long-range connections in the visual cortex selectively link neurons with similar functional properties and this is apparent by their patchiness (Rockland and Lund, 1982; Rockland et al., 1982; Gilbert and Wiesel, 1983; Malach, 1989; Bosking et al., 1997). Although one study reported the intrinsic connectivity of the squirrel visual cortex to show a patchy distribution (Kaas et al., 1989), another account using retrograde tracing shows no evidence for this patchiness (Van Hooser et al., 2006).

These questions support the need for a reassessment of the distribution and retinotopic and functional map organization of the extrastriate visual areas in the squirrel. For the moment, there is no clear evidence that the squirrel might be all that different than other rodents. The null hypothesis would state that the squirrel would have multiple extrastriate areas adjoining V1, each comprising a complete representation of the visual field as in mice. The internal organization would be, as in other rodents, lacking functional maps and with local connection that are not patchy (Burkhalter, 1989; Rumberger et al., 2001).

## On size and connections

It is generally accepted as a clear trend in mammalian brain evolution that greater brain size is correlated with an increase in the number of distinct cortical areas (Campos and Welker, 1976; Kaas, 1987) and increased cortical folding. Several hypotheses have been proposed for mechanisms explaining the appearance in evolution of novel cortical areas. The parcellation theory of Ebbesson (1980), although it has been largely discredited, makes several important observations. In its initial formulation, the theory states that complexity and novel brain structures arise through the parcellation of extant structures and by the selective loss of connections of the novel “daughter aggregates”. The objections to this theory will not be reviewed here but the main problem is the hard stance on the loss of connections as the main mechanisms for novelty and differentiation of brain structures (Striedter, 2004). The interesting aspect of this theory however is the link between divergence of brain areas and connectivity. The parcellation model could predict that increasing the number of cortical areas would lead to more specialized, less globally connected individual areas.

Another hypothesis has been proposed to explain the formation of novel cortical areas by the aggregation and pulling out of cortical modules. One of the key observations towards understanding this model of cortical evolution by modular aggregation is the presence within cortical areas of heterogeneities, modules, that can be distinguished by specific functional and structural properties (Krubitzer and Huffman, 2000). Such modules are exemplified by whisker barrels, blob and interblob patches of the visual cortex of primates, orientation specific columns etc. Krubitzer proposed that these modules could represent intermediate stages in the emergence of a cortical area. These modules would be under two opposing selective pressures. In some instances the element of these modules would aggregate under the pressure to decrease connection length and increase transmission speed, whereas in other circumstances these modules would be pressed to “pull out” of the area where they are located to form a new cortical area (Krubitzer and Huffman, 2000). These two models of cortical arealization both suggest a link between the multiplication of areas and connectivity.

It is further suggested that this pulling out of specific modules would explain the formation of novel cortical areas and the type of connectivity between the areas within the whole network. As in the parcellation hypothesis, the brain would then evolve toward a less global connectivity and greater segregation of modules. One of the main driving forces for this process would be the optimization of the network through the maximization of processing complexity with minimal costs (Ringo, 1991; Ringo et al., 1994; Cherniak et al., 2004; Chklovskii and Koulakov, 2004). This increase in the number of cortical areas through this process is hypothesized to shape the network structure of the cortex (Krubitzer, 2009) in that there are less long range connections and more short connections in larger brain typical of small-world types of networks (Bassett and Bullmore, 2006; Krubitzer, 2009).

**Figure 1:**
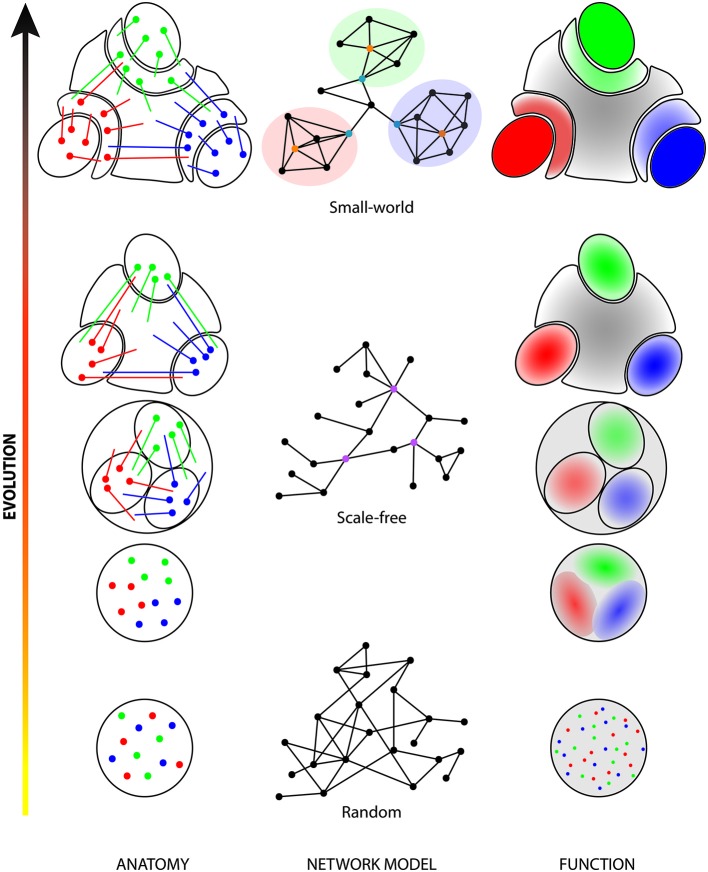
**Evolution of parcellation and network structure of the cerebral cortex**. In early evolutionary stages (bottom row), cells processing different sensory stimuli or different parameters of a stimulus (red, green and blue dots) are intermingled. This random organization directly influences the structure of the network (random network; middle column, first level) and the functional architecture of the area (random distribution). During evolution, neurons of similar functions gathered together (left column) to form functional clusters (right column; shaded red, green and blue zones). Those clusters were initially highly interconnected with each other but, as they were pulled-out of the initial map, their segregation became more and more clear and connections between the clusters became less numerous. This resulted in more functionally homogeneous areas (shaded red, green and blue ovals) separated by areas highly connected with all clusters with heterogeneous properties (gray areas). The high number of connections between different clusters and the presence of several hubs (purple dots) in the network corresponds to a scale-free architecture (middle column, second level). In higher mammals (top row), the initial clusters (plain red, green and blue ovals) are almost completely separated from each other’s and new intermediate secondary sensory areas (shaded red, green and blue crescents) appeared. Those are highly connected with the initial clusters and, together, they now form cortical modules (highlighted areas of the network). Those modules contain provincial hubs (orange dots) that represent areas highly connected with other areas of the same module. Intermediate areas, which are also connected with other intermediate and multisensory areas, can be considered as connector hubs (turquoise dots). This organized structure resulted in the development of the cortical hierarchy and of the small-world network architecture (middle column, third level). In the left column, colored dots are cell bodies and colored lines represent cortical projections. In the middle column, dots are areas and lines are connections between those areas. In the right column, red, blue and green dots or areas indicate different functional properties. Gray color indicates a heterogeneous function.

This proposed model of cortical arealization by modular aggregation and exclusion (see Figure 1) would predict that the initial random cortical map has a low clustering coefficient and low node degrees and thus heterogeneous connections. With increasing complexity, neurons start to connect more with other functionally related neurons. This connectivity model leads to the emergence of the scale-free network architecture characterized by higher node degrees and by the appearance of cortical hubs. As functional subnetworks are regrouping, they are pulled out of the initial map to give rise to specialized areas and more specific modules. This results in a higher clustering coefficient and in a small-world network architecture. One could predict that the cortical areas in the mouse would be more highly interconnected than in primates. A recent network analysis of the visual areas of the mouse supports this prediction (Wang et al., 2012). Indeed although the network of visual areas in the mouse approaches a small-world topology because of the numerous extrastriate areas and the evidence for two functional streams as in primates, each area has a much greater connectivity with all the other areas and most of these connections are reciprocal. This will have important functional consequences on the balance between global synchronization and segregation of modules within the cortical network (see below).

## Salt-and-pepper layout in rodent cortex

The visual cortex in many species is highly segregated in modules that are distinct with respect to their functional properties and connectivity. Typically, there are ocular dominance columns that receive thalamic input from eye specific thalamic geniculate layers. These have been demonstrated quite clearly in Old World monkeys (Hubel and Wiesel, 1968, 1972; LeVay et al., 1985) and more recently in New World monkeys (Markstahler et al., 1998; Fonta et al., 2000; Xu et al., 2005; Kaskan et al., 2007; Takahata et al., 2014). In the primary visual cortex there are cytochrome oxidase (CO) rich blobs and interblobs (Wong-Riley, 1979; Horton and Hubel, 1981) that have specific connectivity with thick stripes and thin stripes of the extrastriate cortex V2 (see references above). Ocular dominance columns and CO blobs are spatially registered in Old World monkeys but not in New World monkeys (Adams and Horton, 2009). In addition, there are functional columns of orientation selectivity in which cells respond to a specific stimulus orientation in primates (Hubel et al., 1978; Blasdel and Salama, 1986), carnivores (Hubel and Wiesel, 1963; Grinvald et al., 1986; McConnell and LeVay, 1986; Rao et al., 1997), ungulates (Clarke et al., 1976) and tree shrew (Humphrey and Norton, 1980; Bosking et al., 1997).

On the other hand, the visual cortex of rodents is organized in what has been coined a salt-and-pepper distribution of cells, without a columnar grouping of cells that share functional properties (see Ohki and Reid, 2007; Kaschube, 2014). Indeed, even if neurons of the visual cortex exhibit specific functional specializations such as orientation selectivity, they show no evidence of structured functional maps in mice (Niell and Stryker, 2008; Van den Bergh et al., 2010), rats (Girman et al., 1999) or even in more visual diurnal and larger brained rodents such as squirrels (Van Hooser et al., 2005a). However, there is recent evidence for ocular dominance domains in the visual cortex of rats (Laing et al., 2014). Such domains have not been shown in other rodents.

There is however some evidence that the output of the visual cortex of the mouse is organized in functionally distinct streams of information. As in monkey, extrastriate areas are organized in dorsal and ventral streams, with anterolateral (AL) and lateromedial (LM) being the two gateways to these pathways, respectively (Wang et al., 2012). Neurons in AL have a greater orientation or direction selectivity and are tuned to lower spatial frequencies than those in anteromedial (AM; Marshel et al., 2011). There are two independent studies that show that extrastriate visual areas receive inputs from functionally distinct neurons of V1 (Glickfeld et al., 2013; Matsui and Ohki, 2013). These selective projections from the primary visual cortex indicates that the parallel processing is starting at least in V1 for these functional properties even though the neurons in the primary visual cortex are not grouped together in functionally homogeneous modules as in monkeys.

It was believed that the brains of mice and rats were too small and that they did not have sufficient visual acuity to require functional maps. The absence of such maps in the squirrels argues against the hypothesis that brain size and higher visual performance are related to the formation of functional maps (Van Hooser et al., 2005a). It has been considered that the columnar organization is not critical for the emergence of the basic functional cell types in the visual cortex such as orientation and direction selectivity (Van Hooser, 2007).

This salt-and-pepper distribution has often been considered as the manifestation of a random organization of close local cortical connections, in agreement with Peters’ rule, which dictates that axons make random connections with dendrites in proportion to their occurrence in the neuropil with no local specificity (see DeFelipe et al., 2002; and Ohki and Reid, 2007 for discussion and references). Although there are some examples which could support a random probabilistic local cortical connectivity (Kalisman et al., 2005), there are several studies demonstrating that the fine local cortical circuitry is highly structured and not a probabilistic function of distance between cells. Indeed, there is evidence for the existence of more highly connected neurons that appear to form structured local subnetworks in the visual cortex of rodents (Song et al., 2005; Yoshimura and Callaway, 2005; Yoshimura et al., 2005). Moreover, at least some subnetworks seem to be related to orientation selectivity (Hofer et al., 2011; Ko et al., 2011). In addition, in the mouse, clonally related neurons have similar orientation selectivity and, even if some do not share this preferred orientation, it suggests that cell lineage is involved in the development of response selectivity and in the determination of the structure of cortical subnetworks (Ohtsuki et al., 2012). These authors suggested that the strong connectivity between sister cells (Yu et al., 2012) establishes a network of neurons that share similar functional properties (Ohtsuki et al., 2012) that could explain the salt-and-pepper organization of the rodent visual cortex. Clonally related neurons share a significant degree of functional properties and neurons of different clones are intermingled in the mouse (Ohtsuki et al., 2012) whereas they undergo less extensive radial dispersion in the monkey (Kornack and Rakic, 1995) and could contribute in the formation of more homogeneous functional columns. However, they note that this explanation is contradicted by the more radially dispersed clonally related neurons in the ferret cortex (Reid et al., 1997). As an alternate scenario, they propose that in species with functional modules in the cortex, each single column could derive from multiple clones and that some mechanisms may act to assemble functionally similar neurons. The initial understanding of the presence of these columns was that they were the result of evolutionary pressure to minimize cortical wiring (Hubel and Wiesel, 1977) and simulations suggest that wiring economy appears as a likely mechanism for grouping of neurons in such columns (Koulakov and Chklovskii, 2001).

### Can the salt-and-pepper layout of mouse cortex be optimal?

Wiring length minimization predicts that a salt-and-pepper layout should yield a connectivity pattern with no preferences for a specific orientation (Chklovskii and Koulakov, 2004; see also Kaschube, 2014 for discussion). However, it has been suggested that orientation selectivity can emerge in a salt-and-pepper distribution of specific functional cell types and a random connectivity between these cells when there is a specific local connectivity in which the large untuned excitatory and inhibitory components balance out (Hansel and van Vreeswijk, 2012).

There is an increasing body of work that supports the idea that there is not one canonical micro-network in the cortex but multiple more or less interrelated and possibly also parallel subnetworks within the visual cortex in rodents. For example, it has been shown that highly interconnected neurons in layers 2–3 are also preferentially connected to a subgroup of layer 4 neurons (Yoshimura et al., 2005). Furthermore, these authors have shown that connections to layers 2–3 coming from layer 5 pyramidal neurons and from layer 2–3 and 4 inhibitory interneurons do not respect these connection defined subgroups, providing opportunities for information exchange between these fine-scale cortical subnetworks (Yoshimura et al., 2005). In addition, they have shown that fast-spiking interneurons establish reciprocal connections with specific subgroups of pyramidal neurons (Yoshimura and Callaway, 2005). There is no simple and general rule of connectivity between neighboring neurons and different connection rules seem to apply to the different subgroups of neurons. For example, there are also specific connectivity patterns within cells of the visual cortex that are related to cortical output streams. Layer 5 pyramidal cells project to several subcortical targets, namely the striatum, superior colliculus and thalamic nuclei. The probability of connections between these output neurons is related to the pre- and postsynaptic target of the neurons. Specifically, the frequency of connection between corticostriatal pyramidal neurons is greater than between corticocortical or corticotectal pyramidal neurons. Moreover corticocortical neurons are more than three times more likely to maintain local connections with neighboring corticotectal pyramids than with any corticocortical or nonadjacent corticotectal pyramids (Brown and Hestrin, 2009).

**Figure 2:**
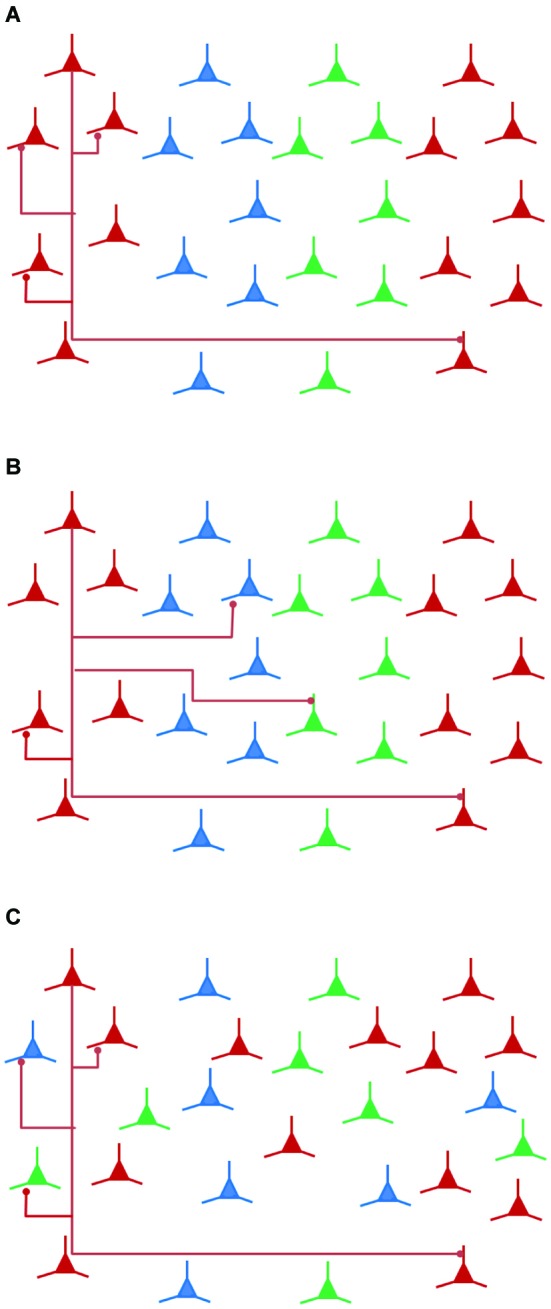
**Local connectivity and wiring economy**. Local connectivity is important in wiring costs optimization. **(A)** Local connectivity within a functionally homogeneous column is also between a homogeneous subgroup of neurons. This configuration shows that functional columns are economical in wiring compared to **(B)**, in which a particular functional class (red neurons) is locally connected to functionally or clonally diverse neurons. **(C)** When local connectivity is heterogeneous, a salt-and-pepper layout of functional categories of neurons offers an economical wiring solution.

If a rule of wiring efficiency or minimization is applied in the formation of columns of functionally similar neurons, this would mean that the wiring costs of one or possibly several subnetworks are limiting factors with possibly increasing brain size. Wiring costs optimization should consider competing costs of local fine scale wiring, local intermodular wiring and also of long distance connectivity (see Figure 2). Simulations strongly suggest the functional maps in the cortex arise for minimizing cost of wiring namely between cells with similar orientation specificities (Koulakov and Chklovskii, 2001).

The salt-and-pepper organization of the rodent cortex could simply be the best available compromise for wiring efficiency for the rodent visual system. There is no reason to believe that there is only one optimal solution that would apply to all subnetworks. Each type of cortical subnetwork is likely under different constraints for efficiency and economy of wiring. The forces at work to bring together functionally related cells in a columnar map seem to have favored orientation selectivity in many cases as in primates, carnivores and tree shrews. These forces could simply be counterbalanced by others that apply to other structural and functional properties within these competing subnetworks, resulting in an intermingling of functionally different neurons even though functionally similar neurons might maintain strong interconnectivity. The identification of connectivity at the single cell level combined with genetic analysis of individual neurons will allow for the identification of the wiring optimization constraints for each of the cortical subnetworks. It is proposed here that the optimization of the wiring between small scale and between mesoscale networks will be instrumental in understanding the origin of the modular organization of the cortex in primates and of the salt-and-pepper layout of neurons in rodents.

There is also evidence suggesting that the wiring economy in rodents and primates brains is not governed by the same rules. The white and gray matter increase in size with respect to the increase in neuronal number in rodents and primates but they scale differently (Ventura-Antunes et al., 2013). Indeed, in primates, the white matter increases at a slower rate than the increase in the number of neurons. As a result, for a given number of cortical neurons, there is a smaller volume of white matter in primates than in rodents (Ventura-Antunes et al., 2013). As pointed out by these authors, there is a decreasing connectivity with growth in small-world networks but the increase in size in rodents results in a constant connectivity fraction as a uniform network would (Ventura-Antunes et al., 2013). This supports the idea that the wiring constraints are different in rodents and primates.

The salt-and-pepper cortex of rodents is not necessarily a simple random or even suboptimal cortical organization, but the expression of constraints different to those of modular cortices. The mouse offers many opportunities for the study of the wiring rules and development of cortical subnetworks with more genetic tools than primates. Investigations at this scale of cortical microcircuitry in primates will be necessary to know what they have in common with the mouse.

## Sensory pathways

The brain of the mouse has fewer cortical areas than primates. However, mice, just like primates, have sensory systems that require several cortical areas to process information from the periphery. The small size of their brain and the fewer cortical areas compared to primates could suggest that either some aspects of the sensory processing are simpler in mice than in primates or that the small size and less differentiated cortex represents the optimal evolutionary solution for the mouse.

### Ascending sensory pathways

It is generally believed that ascending lemniscal sensory pathways are organized in parallel channels reaching the primary sensory cortices from which information is then distributed to more specific cortical networks for further analysis. There is indeed almost no cross talk between sensory pathways except for a few cross-projections in which the inferior colliculus (Tokunaga et al., 1984; Shore et al., 2000; Zhou and Shore, 2004, 2006) and cochlear nuclear complex (Wolff and Künzle, 1997) receive trigeminal afferents. The senses come together nevertheless quite significantly in the superior colliculus, where important multisensory interactions are elaborated (Stein and Meredith, 1993). The multisensory interactions that take place in these layers of the superior colliculus do not give rise to ascending multisensory pathways to the cortex, but rather form descending streams involved in motor pathways for body orientation. As a result, primary sensory cortices receive unisensory ascending projections from specific thalamic nuclei (but see below). Unisensory cortices then give rise to parallel feedforward streams of information processing through cortical networks that eventually reach multisensory processing areas, mainly located in the frontal, temporal and parietal lobes, where unified multisensory percepts are believed to be elaborated for conscious perception and action. Multisensory areas can, in return, send modulatory feedback projections to lower cortical areas.

### Visual streams in the mouse

As in primates, extrastriate areas of mice were shown to be distributed in two functional streams. Anatomical and calcium imaging experiments showed that lateral areas LM, laterointermediate (LI), posterior (P) and postrhinal (POR) project to the ventral stream and that lateral areas AL, rostrolateral (RL) and anterior (A) and medial areas posteromedial (PM) and AM are associated with the dorsal stream (Wang et al., 2011, 2012; Glickfeld et al., 2013). The functional properties of the neurons situated in these extrastriate areas also seem to correspond to what is usually found in primates (Andermann et al., 2011; Marshel et al., 2011; Roth et al., 2012; Glickfeld et al., 2013). The functional properties of extrastriate areas therefore seem to have been either conserved or convergent during evolution, although the properties of the neurons will be fine-tuned to fulfill their role in a way that suits each species (see Huberman and Niell, 2011 for review).

### Cross-modal pathways in primates

There is increasing evidence showing that combining information from the different sensory modalities is important in perception and cognition (Murray and Wallace, 2012; Stein, 2012). In classical models of cortical organization, multisensory integration occurs only in high-order association cortices (Felleman and Van Essen, 1991). In monkeys, several areas of the parietal, temporal and frontal lobes are clearly involved in multisensory processing. Multisensory convergence in the cortex of the superior temporal sulcus (STS) was demonstrated by its responsiveness to visual, auditory and somatosensory stimuli (Desimone and Gross, 1979). The cortical areas of the STS receive visual projections from parietal (Seltzer and Pandya, 1978, 1994) and temporal cortices (Boussaoud et al., 1990; Kaas and Morel, 1993; Saleem et al., 2000), auditory projections from the auditory belt (Morel et al., 1993) and parabelt areas (Seltzer and Pandya, 1978, 1994; Hackett et al., 1998) and somatosensory projections from parietal cortex (Neal et al., 1988; Seltzer and Pandya, 1994; Lewis and Van Essen, 2000). There are several areas in the intraparietal sulcus (IPS) where visual, auditory and somatosensory information converge (Cavada and Goldman-Rakic, 1989; Blatt et al., 1990; Hackett et al., 1998; Beck and Kaas, 1999; Lewis and Van Essen, 2000; Nakamura et al., 2001). It is noteworthy here that these sensory inputs to high order association cortices originate from high order sensory cortices and not from primary sensory cortical areas.

In primates, very few neurons project directly from one primary sensory area to another (Falchier et al., 2002; Clavagnier et al., 2004). In rodents, anatomical evidence revealed multimodal inputs in areas surrounding primary sensory cortices in rats (Paperna and Malach, 1991) and mice (Laramée et al., 2011). In contrast to monkeys there are significant direct cross-modal connections between primary sensory areas in marsupials and rodents. They have been observed in opossums (Kahn et al., 2000; Karlen et al., 2006; Dooley et al., 2013), gerbils (Budinger et al., 2000, 2006, 2008; Henschke et al., 2014), prairie vole (Campi et al., 2010), mice (Wang and Burkhalter, 2007; Charbonneau et al., 2012) and rats (Stehberg et al., 2014). Electrophysiological recordings detected multisensory neurons (suprathreshold response to inputs to more than one sensory modality) in the primary cortices of opossums (Karlen et al., 2006), whereas their incidence was quite low in the center of unisensory cortices of rats but increased in their periphery and in higher areas (Wallace et al., 2004). In monkeys, multisensory neurons (suprathreshold response) were only detected in higher areas (Schroeder et al., 2001; Schroeder and Foxe, 2002; Fu et al., 2003; Ghazanfar et al., 2005; Kayser et al., 2005). What is surprising here is that cross-modal connections in rodents result in multisensory suprathreshold responses in primary sensory cortices, whereas they remain undetected in primates. Only spatially and temporally coherent cross-modal stimuli that result in multisensory integration (see Stein and Stanford, 2008 for review) can functionally reveal cross-modal connections in low order cortical areas in primates (Molholm et al., 2002; Ghazanfar et al., 2005; Lakatos et al., 2007; Kayser et al., 2008). This indicates that feedback cross-modal inputs reaching unisensory cortices in monkeys only have a subthreshold influence on the post-synaptic neurons (Allman et al., 2009). The difference between mice and monkeys regarding the presence or absence or multisensory neurons in low order cortical areas could therefore simply be the consequence of the number and strength of cross-modal inputs reaching these areas.

### Cross-modal pathways in rodents

The presence of quite strong direct cross-modal connections between low order cortical areas in the mouse compared to primates is in agreement with the formation of cortical areas by pulling out of specific functional modules hypothesis. If this is the case, one would therefore expect a higher prevalence of cross-modal connections between primary sensory areas and a higher number of multisensory neurons in areas that are usually considered as unisensory in more primitive mammals. There is indeed a lot of evidence showing that the primary sensory cortices receive more cross-modal projections from other primary sensory cortices in the opossum (Kahn et al., 2000; Karlen et al., 2006; Dooley et al., 2013) and rodents (Budinger et al., 2000, 2006, 2008; Campi et al., 2010; Charbonneau et al., 2012; Henschke et al., 2014) than in primates (Falchier et al., 2002; Clavagnier et al., 2004).

The actual sensory maps in ancestral mammal are not known but it is hypothesized that cross-modal cortical connectivity was greater than in the more derived and segregated cortices (Schneider, 2014). The greater multimodality of the primary sensory cortices in rodents and marsupials would support the idea that the parcellation of unimodal areas from an initial multimodal cortex is incomplete (Schneider, 2014). This does not mean that the rodent cortex is suboptimal, evolution is an ongoing process and each species is a compromise between many competing constraints, but rather that this less segregated state of primary sensory cortices might be, as mentioned earlier, the appropriate adaptive optimum for the behavioral requirements of these animals.

Instead of taking place in very high level temporal and parietal cortices as in primates, multisensory integration in the mouse cortex is achieved in the primary sensory cortices and in the secondary sensory cortices. The greater intermomular connectivity between the visual, somatosensory and auditory cortices (see further) than in primates indicates that these areas of multisensory convergence have not segregated and expanded into the multitude of areas observed in primates. Visual extrastriate areas in the mouse are not unimodal in that they show much evidence for multisensory integration. There are important concentrations of multimodal neurons in the periphery of the primary visual cortex of the rat (Paperna and Malach, 1991). The lateral extrastriate cortex receives direct projections from the primary auditory cortex that terminate on dendrites of neurons that project directly to the primary visual cortex in the mouse (Laramée et al., 2011). The implication of extrastriate areas in multisensory processing is supported by the strong activation of the lateral part of V2 (V2L) following an audio-visual task in the rat (Hirokawa et al., 2008) and by the abundant potential connectivity among multimodal areas surrounding unisensory cortices (Paperna and Malach, 1991). In addition, direct projections from the primary auditory cortex (A1) to V2 have been demonstrated in other rodents such as gerbils (Budinger et al., 2000), prairie voles (Campi and Krubitzer, 2010) and rats (Miller and Vogt, 1984). These projections can further support multisensory processing in V1 through direct feedback connections to V1, which were observed in primates (Rockland and Pandya, 1979; Tigges et al., 1981), tree shrews (Lyon et al., 1998), cats (Squatrito et al., 1981; Symonds and Rosenquist, 1984a,b; Olavarria, 1996) as well as rodents (Olavarria and Montero, 1981, 1989, 1990; Simmons et al., 1982; Coogan and Burkhalter, 1990, 1993). Also, area 2 in mouse, known as the auditory dorsal field, receives projections from auditory, visual and somatosensory cortices as well as from parietal cortices and is clearly involved in multisensory processing (Hishida et al., 2014). Furthermore, a recent study elegantly demonstrated that cross-modal information conveyed by multisensory parietal cortex is implicated in the development of the visual field maps in the primary visual cortex in the mouse (Yoshitake et al., 2013).

The mouse is therefore a very interesting model for the study of cross-modal sensory integration at the level of the primary sensory cortices. These studies are relevant to cross-modal plasticity of the sensory cortices and in this particular case following the loss of vision. Many studies have shown that the visual cortex is activated by other sensory modalities in blind humans (Wanet-Defalque et al., 1988; Kujala et al., 1995a,b, 2005; Sadato et al., 1996; Cohen et al., 1997; Leclerc et al., 2000; Weeks et al., 2000; Burton et al., 2002a,b, 2004, 2006; Burton, 2003; Théoret et al., 2004; Gougoux et al., 2005; Voss et al., 2006, 2008; Weaver and Stevens, 2007; Collignon et al., 2009, 2011). One particular case is of particular significance. It has been demonstrated that in intact sighted human cases, blindfolding induces cross-modal activation of the visual cortex (Pascual-Leone et al., 2005). This demonstrates that there are cross-modal pathways that are functional but possibly silent or subthreshold in the normal visual cortex in humans. Cross-modal pathways in primates and mice are most likely different because, as discussed above, the direct cross-model pathways are more robust in the mouse; but the mouse offers better opportunities than primates to understand these direct routes and their functional significance.

## Cortical hierarchy

Information processing for perception and action appears to require a hierarchical structure of cortical architecture with a dual mode of connectivity between areas by either feedforward or feedback connections. Feedforward and feedback connections are respectively involved in bottom-up and top-down flow of information in the cortex. In primates, feedforward projections arise mostly from supragranular layer 3b, but also from infragranular layer 5, whereas feedback projections originate mainly from infragranular layer 6, but also from layers 2/3a (Rockland and Pandya, 1979; Markov et al., 2014). The laminar distribution of their axon terminals is also distinct; feedforward neurons project onto the granular layer, whereas feedback connections target supragranular and infragranular layers and avoid layer 4 (Rockland and Pandya, 1979). In rodents, feedforward projections arise mostly from supragranular layers and feedback projections mostly originate from infragranular layers. The projection patterns of feedforward connections are quite similar to those found in primates, but the feedforward connections show some differences. In addition to layer 4, feedforward axons in rodents also target the supragranular and infragranular layers (Coogan and Burkhalter, 1990). The difference between feedforward and feedback axonal projections in rodents is therefore the presence or absence of axon terminals in layer 4, respectively.

Bottom-up and top-down pathways allow the identification of the hierarchical relationship between two cortical areas (Rockland and Pandya, 1979; Maunsell and van Essen, 1983; Felleman and Van Essen, 1991; Coogan and Burkhalter, 1993; Scannell et al., 1995). With this organization scheme, the visual system comprises two functional streams with several hierarchical levels in primates (Maunsell and van Essen, 1983; Felleman and Van Essen, 1991; Barone et al., 2000; Vezoli et al., 2004; Markov et al., 2014) and cats (Scannell et al., 1995). A similar organization has also been recently described in mice even if they have fewer cortical areas than primates (Wang et al., 2012). This suggests that, notwithstanding the small size of the brain and the limited number of cortical areas in the mouse, a hierarchical scaffold is still present. Moreover, the ubiquity of the hierarchical organization of the cortex in these diverse animals suggests that it emerged in a quite distant common ancestor, and that it is a very efficient strategy or design for sensory processing.

### Models of cortical organization

The study of the mouse visual cortex from Wang et al. (2012), suggest a similar hierarchical organization in mice and primates, with fewer areas and potentially fewer hierarchical levels in the mouse. This suggests that the rules governing the establishment of cortical circuits have been conserved during evolution. Models have been developed over the years to study how cortical circuits are established in primates, but also in other species. The first evidences suggested that cortical connections depend on the hierarchical relationship between two interconnected areas, with areas or the same hierarchical levels being highly connected. However, further investigations using connectivity matrices revealed that only a small percentage of connections actually fit the hierarchical model (Scannell et al., 1995). This indicated that other factors also participate in the establishment of cortico-cortical connections. Mitchison (1991) proposed that cortico-cortical connections should be organized in a way to optimize cortical wiring in order to limit energy costs. This theory led to the “nearest neighbors” model, which stipulates that adjacent areas are highly connected and distant areas are weakly connected. This model fits quite well with the anatomical evidences from the visual system (Young, 1992) and neocortex (Young, 1993) of primates and the neocortex of cats. The alternate “next-door-neighbor-or next-door-but-one” model proposes that, connections between adjacent areas are strong, those between areas that have few common neighbors are moderate and where those between areas having only one common neighbor are weak. This model was shown to fit better with the connectivity profiles than the nearest neighbor model (Young, 1992; Scannell et al., 1995) and could constitute a trade-off in term of energy and biochemical costs.

Since the years 2000, a new approach has been used to understand how cortico-cortical circuits are established. Instead of looking only at the presence or absence of connections, numbers of projecting neurons with respect to the total number of neurons projecting to the area of interest are now being counted in order to determine the weight, or strength, of the connections (Vezoli et al., 2004). In the macaque visual cortex, connections were found to be very dense between neighboring areas and weaker with more distant areas (Markov et al., 2011). A close relationship between the strength of the connections and the hierarchical distance was also demonstrated (Markov et al., 2014). The study of Markov et al. (2011) also elegantly demonstrated that the density of cortico-cortical connections obey a lognormal distribution spanning across nearly six orders of magnitude, regardless of the cortical areas. Other studies have also found this lognormal organization of cortico-cortical connections with an order of magnitude of 5 in the neocortex of monkeys (Ercsey-Ravasz et al., 2013) and mice (Oh et al., 2014). In the visual system of mice, a lognormal distribution was also found but had a smaller (2–3) order of magnitude (Wang et al., 2012).

The order of magnitude of the lognormal distribution indicates the difference in amplitude between the strength of all possible connections in a system. As mentioned above, the distribution of cortico-cortical connections depends on the physical and hierarchical distances between areas, nearby areas having stronger connections and thus higher connectivity indexes (Markov et al., 2011). In monkeys, the order of magnitude was found to be slightly above 5 for the whole neocortex and visual system. An order of magnitude of 5 was also found in the mouse neocortex, whereas its visual system had an order of magnitude reaching only 2–3, depending on the extrastriate area. The order of magnitude of the neocortex in both mice and primates (order of 5) could indicate that, although mice have a smaller brain size than primates, similar relative physical and hierarchical distances and similar intensity of connections between cortical areas can be found in both species. In the visual system, however, the smaller number of orders of magnitude in the mouse (order of 2–3) compared to primates (order of 5) could indicate that fewer hierarchical steps are involved in visual processing. This would be consistent with the fact that the visual system of rats (and possibly mice) consists of only 3 hierarchical levels (Coogan and Burkhalter, 1993), whereas the visual system of primates has up to 10 levels (Felleman and Van Essen, 1991; Markov et al., 2014). These results also suggest that the visual cortical network in mice is less complex than in primates.

## On complexity

Simplicity or complexity of the brain is not easily defined, and a single metric that can allow a scaling of different species with regards to complexity remains elusive. We will not review here theories on complexity as a very insightful review of the definition of complexity in the brain is provided by Sporns and collaborators (see Sporns, 2011). More specifically, they propose that complexity in brain circuits emerges through the interaction and equilibrium between the functional segregation of defined local areas and the interactions between these areas (Tononi et al., 1994; Sporns, 2011). In neuronal systems, each component should have some distinct functional properties and functional autonomy and these should be linked in such a way that allows for system wide coordination. There is no doubt the brain is composed of functionally segregated subnetworks from levels of organization ranging from cellular to brain-wide systems. The cerebral cortex is typically organized in areas that have distinct functional properties and connections and hence cytoarchitectonic features such as the relative importance of cortical layers. This group proposed a measure of neural complexity that “reflects the interplay between functional segregations and integration within a neural system” (Tononi et al., 1994). In this model (see Figure 1), cortico-cortical connections are links between nodes (cortical areas), which are clustered into modules (e.g., sensory systems). The connections between modules are established by two levels of hubs: connector hubs transfer information between modules and provincial hubs are highly connected with all nodes of the module and with the connector hubs. The complexity of the network will be dependent on the functional and anatomical parcellation of groups of neurons and the connectivity within and between these groups or areas of the cerebral cortex. Small-world architectures are characterized by high node clustering and short path lengths, whereas scale free networks are featured by a small number of highly connected hubs (see Sporns, 2011). In this sense, scale free networks scale lower in modularity and could be less complex that small-world or hierarchical modular networks in which the higher modularity would support greater functional segregation of the nodes. A series of studies by this group showed that greater system complexity arises in hierarchical modular small-world type networks (see Sporns, 2011 for a more complete bibliography).

### Brain networks

The network analyses performed on mouse anatomical data^1^ suggest that the mouse cortex is organized in modules linked by connector hubs, as in primates and exhibits high levels of clustering, as in higher mammals. A small-world architecture is therefore also a feature of the mouse cortical network (Oh et al., 2014; see also Sporns and Bullmore, 2014 for critical comments; Wang et al., 2012). However, whereas cortical networks in cats and macaques (Hilgetag et al., 2000; Sporns et al., 2002) and humans (He et al., 2007; Iturria-Medina et al., 2007, 2008; and see Sporns, 2011 for more complete references) exhibit a clear small-world architecture, with a high clustering, short path lengths and multiple hierarchical levels, there is evidence for high node clustering and hub nodes in mouse cortical networks. This organization is more consistent with a scale-free architecture and the mouse network has therefore been considered intermediate between small-world architecture and scale free architectures (Sporns and Bullmore, 2014).

In the mouse visual system, more specifically, the organization of the network also shows some modularity and some properties of small world networks, but it also, as the whole cortical network, shows less distinct modularity and quite high connectivity between modules, even though some particular areas appear to be positioned to act as hubs for specific pathways (see Wang et al., 2012). There is evidence for functional modules that could correspond to a dorsal and a ventral stream of processing as in primates. There is however a wealth of weak connections both within and between these modules. The abundance of weak intermodular connections has important functional consequences (Goulas et al., 2014). Greater intermodular connectivity increases the global synchronization of the whole network, whereas less intermodular connectivity shifts the dynamic balance toward a greater local network synchronization and functional segregation between modules (Gómez-Gardeñes et al., 2010; Zhao et al., 2011; and see also Goulas et al., 2014 for more discussion). This would indicate that the visual system network in the mouse is based on a similar scaffold as monkeys in being close to a small-world network and having similar two streams of information flow, and would be less functionally segregated than monkeys mainly because of the many weak links between all the network components.

Network analyses of cortical connectivity are largely based on the assumption that the strength of a connection is a function of the number of terminals or synapses in a given connection. This view of an anatomical democracy has been challenged by recent evidence that glutamatergic corticocortcal connectivity is not functionally homogeneous. Indeed, studies have shown functional classes of glutamatergic postsynaptic responses that appear to be correlated with presynaptic terminal size (Covic and Sherman, 2011). Moreover, these authors define functional classes in which corticocortical class 1B connections terminate on postsynaptic sites with ionotropic receptors whereas type 2 corticocortical connections terminate on postsynaptic sites with metabotropic receptors (Covic and Sherman, 2011; De Pasquale and Sherman, 2011, 2013). This functional heterogeneity strongly suggests that not all cortical contact exert the same influence on postsynaptic neurons. Network analyses based only on terminal or neurons number might not provide a sufficient overview for understanding the functional architecture of cortical connectivity.

## Concluding remarks

While primates evolved to become large animals with large brains, mice remained small and so did their brain. The mouse brain has both similarities and differences with the primate brain. It is different in that it has fewer cortical areas with fewer visual areas and extensive cross-modal and intermodular cortical connections. Ocular dominance columns are also lacking and, instead, a salt-and-pepper organization is found in mouse visual cortex. Moreover, the brain of the mouse and primates share a similar hierarchical organization based on largely reciprocal feedforward and feedback connections. In addition, cortical connectivity follows similar distance rules in that close areas are more strongly interconnected than distant areas. The visual cortical areas of mice and primates are also similar in that the extrastriate areas are distributed in two functional streams that share many similar functional properties.

Overall, these features show that although the mouse brain and primate differ in absolute and relative size, in the number of hierarchical levels and in the diversity of cortical areas and their modular parcellation, several key features are shared between these animals. Cortical connections develop according to similar wiring rules even though the optimal solutions for wiring economy appear to be different. In the visual system, extrastriate areas are organized in similar functional streams even though the primary visual cortex exhibit very different modular organizations in mice and primates.

## Conflict of interest statement

The authors declare that the research was conducted in the absence of any commercial or financial relationships that could be construed as a potential conflict of interest.

## Footnotes

^1^The connectome of the mouse is being produced by several endeavors such as the Allen Brain Atlas (http://www.brain-map.org/), the Brain Architecture Project (http://brainarchitecture.org/) and the Mouse Connectome Project (http://www.mouseconnectome.org/). The Allen Brain Atlas and the Brain Architecture Project are also working on the connectome of other species.
